# Occurrence of Substituted *p*-Phenylenediamine Antioxidants and Their Quinone Derivatives in the Pearl River Estuary Water System, China

**DOI:** 10.3390/toxics14050356

**Published:** 2026-04-23

**Authors:** Yihao Yin, Binbin Deng, Wenzi Tu, Yongtong Guo, Lixian Chen, Yongjin Liang, Yanlong Zhao, Shaoxian Du, Yi Li

**Affiliations:** 1 Eco-Environmental Monitoring and Research Center, Pearl River Valley and South China Sea Ecology and Environment Administration, Ministry of Ecology and Environment, Guangzhou 510611, China; yinyh6@mail2.sysu.edu.cn (Y.Y.); 19128904522@163.com (B.D.); tuwenzi@zjnhjg.mee.gov.cn (W.T.); guoyongtong@zjnhjg.mee.gov.cn (Y.G.); clx@zjnhjg.mee.gov.cn (L.C.); liangyongjin@zjnhjg.mee.gov.cn (Y.L.); zjly_qc@sina.com (Y.Z.); 2 School of Chemistry, Sun Yat-sen University, Guangzhou 510006, China

**Keywords:** *para*-Phenylenediamine, *para*-Phenylenediamine quinone, PPD, PPD-Q, Pearl River Estuary

## Abstract

Substituted *p*-phenylenediamines (PPDs) and their quinone derivatives (PPD-Qs) are emerging contaminants associated with tire-related antioxidants and antiozonants and have raised increasing concern because of their potential environmental effects. However, information on their occurrence in estuarine systems, particularly their combined distribution in water and sediment, remains limited in the Pearl River Estuary. In this study, 30 water samples and five sediment samples collected from the Pearl River Estuary were analyzed for selected PPDs and PPD-Qs. Four target compounds were detected in water, whereas nine were found in sediments, indicating broader occurrence in the sediment phase. The total concentration of PPDs ranged from below the detection limit to 17.6 ng/L in water and from 0.140 to 1.37 ng/g in sediments. In water, 6PPD and 6PPD-Q showed relatively high detection frequencies, while elevated IPPD concentrations were observed at several sites near urbanized coastal areas. In sediments, the highest ΣPPDs level was recorded in Shenzhen Bay. The observed spatial patterns suggest that mixed anthropogenic inputs may influence the occurrence of these compounds in the estuary, although direct source attribution requires further investigation. Overall, this study provides preliminary baseline information on the occurrence and phase-specific distribution of PPDs and PPD-Qs in the Pearl River Estuary and supports future investigations into their environmental fate and ecological relevance.

## 1. Introduction

Substituted *para*-Phenylenediamines (PPDs) are a series of synthetic antioxidants and antiozonants that are widely used in the rubber industry, mostly for the production of tires [1,2,3]. The use of vehicles causes considerable amounts of tire wear particles, including PPDs and their derivatives, releasing into the environment through the friction between the vehicle tires and road surfaces [4,5]. According to the statistics of 2013 and 2014, the tire wear particle emission release was estimated to be 1.52 million t/year in the USA, 1.33 million t/year in the EU, and 0.76 million t/year in China [6,7].

*para*-Phenylenediamine quinones (PPD-Qs) are a newly discovered class of oxidation or ozonation products derived from PPDs [8]. PPDs and PPD-Qs are both contaminants of emerging concern, with acute toxic effects on several aquatic species [9,10], while inhibiting proliferation and promoting apoptosis in human cells [11]. Substantial studies have reported the occurrence of PPDs and PPD-Qs in various environmental matrices, including soil [12,13], dust [1,14], air [15,16], water [12,17,18], sediment [19,20] and human urine [21,22,23].

Although PPDs and PPD-Qs have been reported in a range of environmental matrices, knowledge of their occurrence in the Pearl River Estuary is still limited, particularly regarding their co-distribution in water and sediment. To address this gap, we collected water and sediment samples from the Pearl River Estuary and analyzed them for selected PPDs and their quinone derivatives. Specifically, this study aimed to examine their occurrence and spatial distribution, compare phase-specific compositional profiles, and assess the potential sources and environmental implications of these contaminants in an important estuarine environment. The results provide a preliminary regional baseline for future studies on the sources, fate, ecological relevance, and management of PPD-related contaminants.

## 2. Materials and Methods

### 2.1. Chemicals and Reagents

Seven PPDs, including *N*-(1,3-dimethylbutyl)-*N*’-phenyl-*p*-phenylenediamine (6PPD), *N*-isopropyl-*N*’-phenyl-*p-*phenylenediamine (IPPD), *N*-cyclohexyl-*N*’-phenyl-*p*-phenylenediamine (CPPD), *N*,*N*’-bis(1,4-dimethylpentyl)-*p*-phenylenediamine (77PD), *N*,*N*’-diphenyl-*p*-phenylenediamine (DPPD), *N*,*N*’-di-2-naphthyl-*p*-phenylenediamine (DNPD), *N*,*N*’-bis(methylphenyl)-*p*-benzenediamine (DTPD), and their quinones (6PPD-Q, IPPD-Q, CPPD-Q, 77PD-Q, DPPD-Q and DTPD-Q), were chosen as target compounds in the present study. ^13^C_6_-6PPD-Q was applied as the surrogate standard, while ^13^C_12_-6PPD-Q and benzophenone-*d*_10_ were used as internal standards. 6PPD, IPPD, DPPD and 77PD were obtained from AccuStandard (New Haven, CT, USA). 6PPD-Q, ^13^C_6_-6PPD-Q and ^13^C_12_-6PPD-Q were purchased from Cambridge Isotope Laboratories (Andover, MA, USA). CPPD-Q and 77PD-Q were acquired from Zzstandard (Shanghai, China). DTPD, DPPD-Q and DTPD-Q were bought from ScrStandard (Shanghai, China). CPPD, DNPD, IPPD-Q and benzophenone-*d*_10_ were obtained from Chemservice (West Chester, PA, USA), Tan-Mo Technology (Changzhou, China), Dr. Ehrenstorfer GmbH (Augsburg, Germany) and Toronto Research Chemicals (Toronto, ON, Canada), respectively. Detailed information of these compounds is listed in Table S1. Primary and secondary amine (PSA) (CNWBOND, 40–63 µm) and C18 absorbent (CNWBOND, 40–63 µm) were purchased from Anpel Laboratory Technologies (Shanghai, China). Hexane, dichloromethane, methanol and acetonitrile were all high-performance liquid chromatography (HPLC) grade (OCEANPAK, Gothenburg, Sweden). The target analytes were selected to represent commonly studied PPD antioxidants used in rubber products and their major quinone derivatives that have been increasingly detected in environmental samples [1,2,3]. In addition, the availability of authentic standards was considered to ensure robust instrumental identification and quantification. Therefore, seven parent PPDs and six PPD-Qs were included in the present study.

### 2.2. Sample Collection

**Figure 1:**
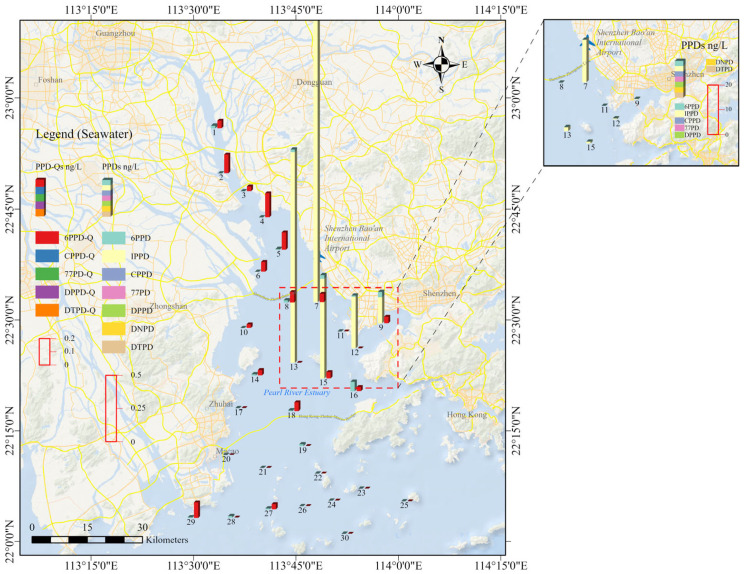
Sampling sites and corresponding concentrations of PPDs and PPD-Qs in estuary water.

The study area is located in the Pearl River Estuary in the Guangdong province of China, and the detailed information of the sampling sites is shown in Figure 1. The sampling design gave priority to broad spatial coverage of the water phase across the estuarine system because water was considered the most responsive matrix for reflecting recent inputs and short-term spatial variability of PPDs and PPD-Qs in this dynamic environment. Accordingly, 30 water samples were collected from the main estuarine zones. Sediment sampling was conducted at five representative sites to enable a preliminary comparison of the occurrence and composition of target analytes between water and sediment. Although the sediment dataset provided useful phase-specific information, its relatively limited spatial coverage may constrain the representativeness and statistical robustness of sediment-related interpretations. Therefore, the sediment results should be considered preliminary and interpreted with appropriate caution.

A total of 35 samples of water (*n* = 30) and sediments (*n* = 5) were collected from the water system, utilizing pre-cleaned brown glass bottles for water samples and Teflon bottles for sediment samples. All the collected water samples were immediately adjusted to pH 2.0 with 4 M H_2_SO_4_, and 5% methanol (*v*/*v*) was added in order to inhibit microbial growth. The water and sediment samples were stored in cold ice boxes and then shortly transported to a refrigerated compartment in the laboratory (4 °C) before processing. All water and sediment samples were extracted within 48 h, and analyzed within 7 d.

### 2.3. Sample Extraction

Processing and treatment of water and sediment samples followed the published approaches with modifications [3,24]. Compared with recent analytical workflows developed for PPDs and PPD-Qs in wastewater, receiving waters, and particulate/solid matrices, the present procedure below was adapted for estuarine samples by integrating the dissolved and particulate fractions of each water sample and applying a unified cleanup workflow to both water and sediment extracts, so as to better support whole-water assessment and phase comparison in the Pearl River Estuary. An amount of 1.0 L of water sample was filtered with glass fiber filter membranes (47 mm, 1.2 μm, Whatman GF/C, Hillsboro, OR, USA). The particulate phase of the water sample was ultrasonicated twice in 10 mL acetonitrile for 15 min, and twice with 10 mL *n*-hexane/dichloromethane (*v*/*v* = 1:1) for 15 min. The extracts were concentrated to 1 mL under nitrogen and combined with the corresponding dissolved phase of water sample. The water sample including dissolved and particulate phases was spiked with surrogate standard (20 ng, ^13^C_6_-6PPD-Q) and processed by solid phase extraction on an Oasis HLB cartridge (200 mg, 6 cc Vac Cartridge, Waters). The HLB cartridge was preconditioned with 10 mL methanol and 10 mL Milli-Q water. Then, the water sample was loaded on the HLB cartridge (flow rate: 5 mL/min), dried under high vacuum for 20 min, and eluted with 10 mL methanol. The eluates were dried under a gentle nitrogen stream and then redissolved in 200 μL of acetonitrile containing internal standards (20 ng ^13^C_12_-6PPD-Q and 20 ng benzophenone-*d*_10_). The samples were finally filtered through 0.22 μm membranes before instrumental analyses.

Sediment samples were freeze-dried, ground and passed through 80-mesh sieves. Next, 3.0 g sediment samples were spiked with surrogate standard (20 ng, ^13^C_6_-6PPD-Q) and then ultrasonicated once with 20 mL acetonitrile for 15 min and twice in 20 mL *n*-hexane/dichloromethane (*v*/*v* = 1:1) for 15 min. The extracts were combined, concentrated to near dryness, redissolved in 0.5 mL methanol and 50 mL Milli-Q water, and purified on Oasis HLB cartridges. The subsequent treatment process was the same as water samples.

### 2.4. Instrumental Analysis

Seven PPDs and their quinones were separated and analyzed utilizing the Xevo TQ-S UPLC-MS/MS system (Waters, Milford, MA, USA), combined with an XBridge BEH C8 column (Waters, 2.5 µm, 2.1 × 100 mm). The mobile phases were 0.1% formic acid in water and 0.1% formic acid in methanol respectively for A and B (flow rate: 300 µL/min). The solvent composition was initially 98% A, gradually ramped to 99% B in 12.25 min and held for 2.75 min, and then rapidly returned to the initial solvent within 0.1 min (held for 2 min). The injection volume for samples was 1 µL. The analytical parameters for target compounds are listed in Table S2, referring to previous studies [16,19,25,26].

### 2.5. Quality Assurance and Quality Control

Field blanks (*n* = 3) and procedure blanks (*n* = 3) were processed successively for each batch of samples. Field blanks containing deionized water were carried to the Pearl River Estuary during sampling, returned to the laboratory, treated identically as the samples, and compared with deionized water stored in the laboratory. All PPDs and PPD-Qs were not detected in the field blanks and the procedure blanks. PPDs and PPD-Qs were spiked into solutions and matrices to assess their recoveries throughout the entire analytical procedure. The mean recoveries of ^13^C_6_-6PPD-Q in water (*n* = 30) and sediment (*n* = 5) samples reached 78.8 ± 5.0% and 85.0 ± 6.8%, respectively. Eleven-point calibration curves spanning 0.025 to 200 ng/mL were applied to quantify the concentrations of PPDs and PPD-Qs, and the regression coefficients surpassed 0.99. The instrument detection limit (IDL) was established as the concentration that has a response with a signal-to-noise (S/N) ratio of five. The method detection limits (MDLs) for 6PPD and IPPD were calculated as the average concentrations plus their triple standard deviation in the procedural blanks. For other compounds, MDLs were defined as the concentrations that generate a response with an S/N ratio of five in matrices. The apparently non-uniform definition of MDLs reflects differences in blank responses among analytes: for 6PPD and IPPD, MDLs were estimated from procedural blank levels, whereas, for analytes not detected in blanks, MDLs were established using a signal-to-noise ratio of five in matrix extracts. The IDLs and MDLs for PPDs and PPD-Qs are displayed in Table S3.

### 2.6. Data Analysis

The correlations in concentrations among diverse PPDs and PPD-Qs in water or sediment samples from the Pearl River Estuary were evaluated employing Spearman’s correlation analysis. The differences among concentrations of various PPDs and PPD-Qs in water or sediment samples were assessed by applying the Mann–Whitney U test. For spatial comparison of the water-phase data, the 30 sampling sites were grouped into three geographic zones according to their location and surrounding anthropogenic characteristics: the northern river–outlet zone (sites 1–6), the inner estuary urban–port–channel convergence zone (sites 7–16), and the outer estuary/offshore mixing zone (sites 17–30). For statistical testing, sites 7–16 were further treated as urban–port-associated inner estuary sites and compared with the remaining sites (1–6 and 17–30) using the Mann–Whitney U test. Statistical significance in difference was set at *p* < 0.05 (two-tailed). Concentrations below the MDL were substituted with half the MDL value for statistical analysis.

## 3. Results

### 3.1. Occurrence of PPDs and PPD-Qs in Water

Four target analytes, namely 6PPD, 6PPD-Q, IPPD, and IPPD-Q, were detected in water samples from the Pearl River Estuary, with detection frequencies ranging from 16.7% to 53.3% (Table 1 and Figure 1). The total concentration of PPDs (ΣPPDs) was in the range of <MDL–17.6 ng/L, with a mean concentration of 0.740 ng/L. Among 13 target PPDs and PPD-Qs, 6PPD-Q was most prevalent in water, with a 53.3% detection frequency, followed by 6PPD (50.0%). Although IPPD was detected less frequently (16.7%), it showed the highest mean concentration in water (0.672 ng/L), mainly because of elevated concentrations observed at sites 7 and 13 (17.3 and 1.58 ng/L, respectively). In addition, a significant positive correlation was observed between IPPD and IPPD-Q concentrations in water (Spearman’s correlation coefficient, *r_s_* = 0.77; *p* < 0.01), indicating a site-specific association between the parent compound and its quinone derivative.

To further examine the spatial pattern of water contamination, concentrations were compared between the inner estuary urban–port-associated sites (sites 7–16) and the remaining sites. IPPD and IPPD-Q concentrations were significantly higher in sites 7–16 than in the other sites (mean: 1.995 vs. 0.010 ng/L for IPPD, *p* < 0.05; mean: 0.047 vs. 0.004 ng/L for IPPD-Q, *p* < 0.05). In addition, the detection frequencies of IPPD and IPPD-Q were both 40.0% in sites 7–16, compared with 5.0% in the remaining sites. By contrast, ΣPPDs, 6PPD, and 6PPD-Q did not differ significantly between the two groups (*p* > 0.05).

### 3.2. Occurrence of PPDs and PPD-Qs in Sediment

**Figure 2:**
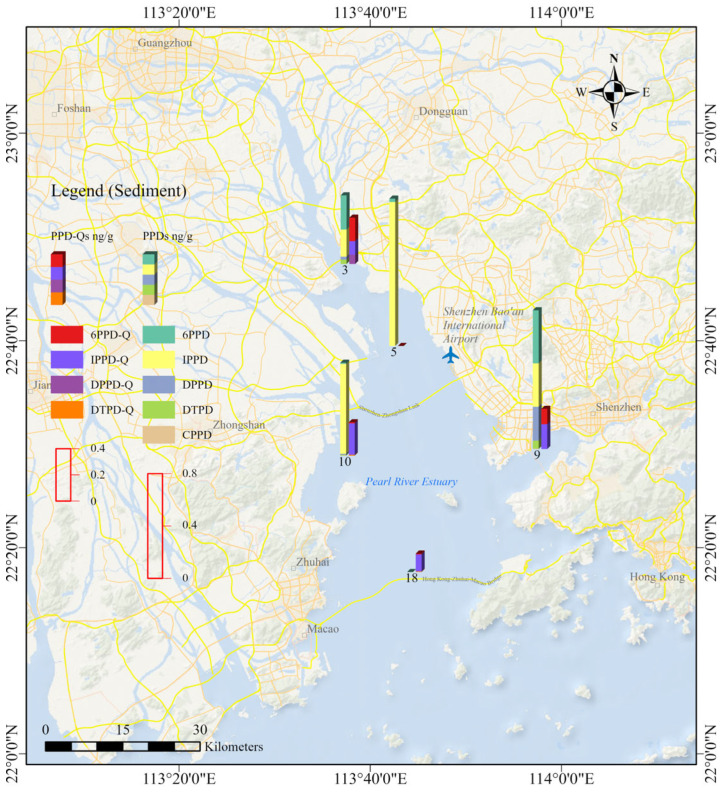
Sampling sites and corresponding concentrations of PPDs and PPD-Qs in estuary sediments.

**Figure 3:**
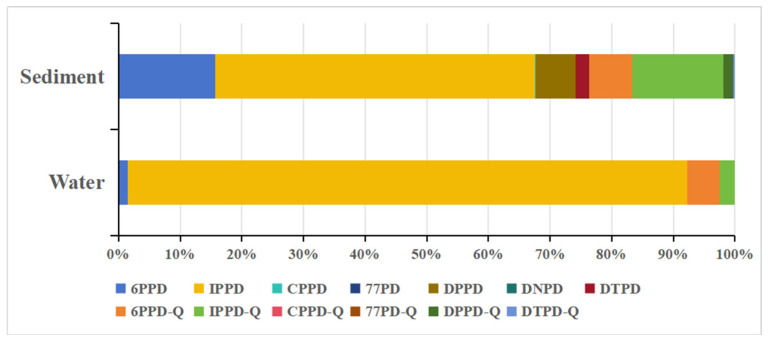
Concentration profile of PPDs and PPD-Qs in water and sediment samples from the Pearl River Estuary.

Nine target PPDs and PPD-Qs were detected (detection frequency 20.0–80.0%) in sediment samples from the Pearl River Estuary (Table 2 and Figure 2). 6PPD, 6PPD-Q, IPPD, IPPD-Q, DPPD, DTPD and DTPD-Q were frequently detected (detection frequency > 60.0%). Sediment concentrations of ΣPPDs were in the region of 0.140–1.37 ng/g (mean 0.897 ng/g). Among the detected analytes, IPPD was the predominant congener (Figure 3), with a mean concentration of 0.465 ng/g (range < MDL–1.10 ng/g), followed by 6PPD (mean 0.141 ng/g, <MDL–0.406 ng/g) and IPPD-Q (0.133 ng/g, <MDL–0.241 ng/g). Because only five sediment samples were collected, these results provide preliminary information on the occurrence and composition of PPDs and PPD-Qs in the sediment phase and should be interpreted with caution.

## 4. Discussion

### 4.1. Occurrence and Comparison of PPDs and PPD-Qs in Estuarine Water

The present results show that PPDs and PPD-Qs were detectable in both water and sediment from the Pearl River Estuary, but their occurrence patterns differed between the two phases. In water, only four analytes were detected, whereas nine compounds were found in sediment, indicating broader occurrence in the particulate-associated phase. A clear spatial pattern was observed in the water samples, with relatively higher ΣPPDs concentrations at sites 7, 9, 12, 13, and 15, which are located near the highway–port–channel convergence zone on the northern and western shores of Lingdingyang. This pattern suggests that the measured concentrations were influenced by mixed anthropogenic inputs in densely urbanized coastal areas rather than by a single uniform source.

To further interpret these water-phase hotspots, potential source pathways should be considered cautiously. Urban roadway runoff appears to be one plausible contributor, because previous studies have repeatedly reported the occurrence of PPDs and PPD-Qs in urban runoff, road dust, and runoff-affected aquatic environments. In particular, stormwater and runoff from traffic-impacted functional areas have been recognized as important transport pathways for tire-related chemicals into receiving waters [4,12,18]. In the present study, however, no direct source-tracing indicators, runoff samples, or traffic-intensity data were collected. Therefore, the association with roadway runoff should be regarded as a plausible explanation for the observed spatial pattern, rather than direct proof of source attribution.

### 4.2. Potential Influence of Port-Related and Coastal Human Activities

In addition to roadway-related inputs, the elevated sites were also situated in areas influenced by port activities, dock operations, and intense coastal human activity. This suggests that port zones may receive multiple overlapping rubber-related inputs. PPDs are not restricted to vehicle tires and may also originate from other rubber-based products used in coastal and port environments [20]. Accordingly, the observed water-phase pattern may reflect the combined influence of urban runoff and port-related activities.

Nevertheless, this inference remains tentative. Because no harbor runoff, dockside material, or vessel-related samples were collected, the possible contribution of maritime activities cannot be separated from co-occurring urban inputs at this stage. Thus, the present results support only a preliminary source interpretation based on spatial coincidence, and direct evidence from source-oriented sampling will be needed in future work.

### 4.3. Comparison with Other Aquatic Systems and Implications for Localized Inputs

Beyond the within-estuary spatial pattern, the measured concentrations also provide context for comparison with other aquatic systems. Concentrations of PPDs and PPD-Qs in the Pearl River Estuary (mean < MDL–0.672 ng/L) were lower than that reported for Jiaojiang River (<MDL–12 ng/L) in Taizhou City, China [20]. The lower levels of PPDs and PPD-Qs in the Pearl River Estuary are primarily attributable to its vast watershed dilution capacity, contrasting sharply with the Jiaojiang River’s confined basin and proximity to concentrated industrial sources in Taizhou City.

Water concentrations of 6PPD and 6PPD-Q detected in this study (mean 0.011–0.039 ng/L) were relatively lower than those reported for Zhujiang River (mean 0.56–2.34 ng/L) and Dongjiang River (0.47–1.69 ng/L) [4]. However, water concentrations of IPPD detected in this study (mean 0.672 ng/L) reached higher levels than upstream surface water (Liuxi River, mean 0.31 ng/L) in Guangzhou, China [27]. These differences suggest that the occurrence of individual PPD-related compounds may vary considerably among aquatic systems depending on local source composition, hydrodynamic conditions, and dilution processes.

In the present estuary, the relatively low levels of 6PPD and 6PPD-Q may reflect dilution and mixing in the open estuarine environment, whereas the elevated IPPD concentrations at a few sites suggest localized inputs that deserve further investigation. The positive correlation between IPPD and IPPD-Q in water is consistent with a site-specific association between the parent compound and its quinone derivative, but this relationship alone does not demonstrate in situ transformation. Additional process-based evidence, such as precursor–product ratios under controlled environmental gradients or time-resolved observations, would be needed to support that interpretation more directly.

### 4.4. Sediment Occurrence and Possible Accumulation Processes

While the water-phase data mainly reflect recent and mobile inputs, the sediment data provide complementary information on retention and redistribution within the estuarine system. The highest sediment ΣPPDs concentration was observed at site 9 in Shenzhen Bay, an area adjacent to the highly urbanized Shenzhen–Hong Kong region. One possible explanation is that this semi-enclosed embayment receives sustained land-based inputs and favors pollutant accumulation because of weaker hydrodynamic exchange and enhanced deposition. This interpretation is consistent with broader evidence showing that estuarine and coastal sediments can act as repositories for PPDs and PPD-Qs transported from urban rivers and nearshore environments [19].

At the same time, sediment concentrations of PPDs and PPD-Qs detected in this study (median < MDL–0.331 ng/g) were relatively lower than those reported in sediments from Pearl River (1.3–5.6 ng/g) and the Pearl River Estuary (<MDL–2.4 ng/g) [19], suggesting that the Pearl River Estuary may currently represent a relatively low-to-moderate contamination setting for these compounds. However, this comparison should be interpreted with caution because sediment concentrations can be influenced by sediment properties, organic carbon content, hydrodynamic conditions, and sampling design.

### 4.5. Water–Sediment Linkages and Phase-Specific Environmental Behavior

A key question arising from the paired water–sediment observations is how these compounds are redistributed between phases. The absence of significant correlations between most parent PPDs and their quinone derivatives in sediment should not be overinterpreted. Previous paired water–sediment studies have shown that detected PPDs and PPD-Qs can exhibit markedly different sediment–water partitioning behavior, with reported mean log*K*_oc_ values spanning approximately 2.0–4.0 among congeners, indicating that their retention and deposition potentials differ substantially [20,28]. Such differences imply that individual compounds may differ in sorption affinity, transport efficiency, and sediment retention potential.

In addition, large-scale surveys have demonstrated that PPDs and PPD-Qs can be transported from urban rivers to estuarine and coastal sediments, suggesting that hydrodynamic transport and land-to-sea redistribution may further reshape sediment profiles during environmental migration [19]. Therefore, the weak correlations observed in the present sediments may reflect the combined influence of differential partitioning, transport, and post-depositional transformation, although these processes were not directly resolved in this study.

The broadly similar composition patterns observed in water and sediment, both characterized by the prominence of IPPD and IPPD-Q, suggest that the two phases may have been influenced by overlapping source mixtures or linked exchange processes. However, similarity in composition alone does not demonstrate that sediment contamination primarily originated from the overlying water. Previous paired water–sediment studies more commonly reported 6PPD and 6PPD-Q as dominant compounds, and the relative abundance patterns of individual congeners can vary across systems depending on source composition and sediment–water partitioning behavior [20]. Accordingly, the present results are better interpreted as preliminary evidence of phase-linked contamination patterns, rather than direct proof of source transfer from water to sediment.

### 4.6. Screening-Level Ecotoxicological Context

Taken together, these occurrence patterns also raise the question of ecological relevance. To place the measured water concentrations in a toxicological context, the detected levels of 6PPD and 6PPD-Q in the present study were compared with currently available aquatic effect thresholds. The maximum concentrations of 6PPD (0.072 ng/L) and 6PPD-Q (0.180 ng/L) were well below the U.S. EPA acute freshwater aquatic life screening values of 8900 ng/L for 6PPD and 11 ng/L for 6PPD-Q, respectively [29,30]. The observed maximum 6PPD-Q concentration was also markedly lower than the revised 24 h LC_50_ of 95 ng/L for coho salmon reported by Tian et al. [11], and lower than the 41 ng/L LC_50_ reported for early life-stage coho salmon by Lo et al. [31], suggesting a low likelihood of immediate acute risk from 6PPD-Q in the sampled waters under the conditions captured by this survey.

However, this comparison should be regarded only as a screening-level assessment, because the currently available benchmark values are mainly based on single-compound acute toxicity data from freshwater species, whereas the present study was conducted in an estuarine system. In fact, the U.S. EPA noted that chronic freshwater values as well as acute and chronic estuarine/marine screening values for 6PPD-Q could not yet be derived because of insufficient data, and comparable limitations also apply to 6PPD [29,30]. In addition, aquatic threshold values remain scarce for IPPD, IPPD-Q, and most other PPD analogs, and sediment quality benchmarks for PPDs and PPD-Qs are still limited [8]. Therefore, although the measured concentrations in water and sediment appear to indicate relatively low immediate risk, the present data should be viewed primarily as regional baseline occurrence information rather than a definitive ecological risk assessment, and further work is needed to establish phase-specific and estuarine-relevant toxicity thresholds.

### 4.7. Implications and Study Limitations

Overall, this study provides initial insight into the occurrence, spatial distribution, and phase-specific profiles of PPDs and PPD-Qs in the Pearl River Estuary. The results suggest that localized urbanized coastal zones may represent important input areas, while sediment may act as a receiving compartment for a broader range of PPD-related contaminants.

At the same time, several limitations should be acknowledged. In particular, the limited number of sediment samples constrains the spatial representativeness and statistical robustness of sediment-related interpretations, and the absence of direct source-tracing information limits confident source apportionment. Future work should therefore combine denser sediment sampling, direct runoff and port-related sampling, and estuarine-relevant toxicity data to better resolve the sources, transport processes, and ecological significance of PPDs and PPD-Qs in this estuarine environment.

## 5. Conclusions

This study provides preliminary baseline information on the occurrence and phase-specific distribution of PPDs and PPD-Qs in the Pearl River Estuary. Four target compounds were detected in water, whereas nine were detected in sediment, indicating broader occurrence in the sediment phase. In water, 6PPD, 6PPD-Q, IPPD, and IPPD-Q were the major detected analytes, and relatively elevated concentrations were observed at several sites located in urbanized coastal zones. These spatial patterns suggest that mixed anthropogenic inputs, including roadway runoff and port-related activities, may influence the occurrence of PPD-related contaminants in the estuary, although direct source attribution was beyond the scope of the present study.

Compared with water, sediment contained a greater variety of target compounds and showed localized enrichment, particularly in Shenzhen Bay, suggesting that sediment may act as an important receiving compartment for PPDs and PPD-Qs in the estuarine environment. The broadly similar composition patterns observed in water and sediment imply possible phase linkage or overlapping source inputs; however, the present dataset does not provide direct evidence that sediment contamination primarily originated from the overlying water. Likewise, the positive correlation between IPPD and IPPD-Q in water indicates a site-specific association between the parent compound and its quinone derivative, but it should not be interpreted as definitive proof of in situ transformation.

A brief screening-level comparison with currently available aquatic effect thresholds suggests a low likelihood of immediate acute risk from 6PPD and 6PPD-Q under the conditions captured by this survey. However, this assessment remains preliminary because toxicological benchmarks for most PPD analogs are still scarce, and estuarine- and sediment-specific threshold data remain limited. In addition, the relatively small number of sediment samples constrains the spatial representativeness of the sediment dataset. Therefore, future studies should incorporate denser sediment sampling, source-oriented investigations, and estuarine-relevant toxicity data to better resolve the sources, environmental behavior, and ecological significance of PPDs and PPD-Qs in the Pearl River Estuary.

## Figures and Tables

**Table 1:** Concentrations (ng/L) of PPDs and PPD-Qs in water samples (*n* = 30) from Pearl River Estuary.

Compound	Detection Frequency	Mean	Median	Range
6PPD	50.0	0.011	0.003	<MDL–0.072
IPPD	16.7	0.672	<MDL	<MDL–17.3
CPPD	0	<MDL	<MDL	/
77PD	0	<MDL	<MDL	/
DPPD	0	<MDL	<MDL	/
DNPD	0	<MDL	<MDL	/
DTPD	0	<MDL	<MDL	/
6PPD-Q	53.3	0.039	0.030	<MDL–0.180
IPPD-Q	16.7	0.018	<MDL	<MDL–0.212
CPPD-Q	0	<MDL	<MDL	/
77PD-Q	0	<MDL	<MDL	/
DPPD-Q	0	<MDL	<MDL	/
DTPD-Q	0	<MDL	<MDL	/

**Table 2:** Concentrations (ng/g) of PPDs and PPD-Qs in sediment samples (*n* = 5) from Pearl River Estuary.

Compound	Detection Frequency	Mean	Median	Range
6PPD	80.0	0.141	0.026	<MDL–0.406
IPPD	80.0	0.465	0.331	<MDL–1.10
CPPD	40.0	0.001	<MDL	<MDL–0.003
77PD	0	<MDL	<MDL	/
DPPD	60.0	0.058	0.008	<MDL–0.260
DNPD	0	<MDL	<MDL	/
DTPD	60.0	0.020	0.005	<MDL–0.062
6PPD-Q	80.0	0.062	0.009	<MDL–0.179
IPPD-Q	80.0	0.133	0.130	<MDL–0.241
CPPD-Q	0	<MDL	<MDL	/
77PD-Q	0	<MDL	<MDL	/
DPPD-Q	20.0	0.014	<MDL	<MDL–0.071
DTPD-Q	60.0	0.003	<MDL	<MDL–0.007

## Data Availability

The original contributions presented in this study are included in the article/Supplementary Materials. Further inquiries can be directed to the corresponding authors.

## Supplementary Materials

The following supporting information can be downloaded at: https://www.mdpi.com/article/10.3390/toxics14050356/s1, Table S1: Full names, CAS numbers, molecular formulas, structures and physicochemical properties of PPDs and PPD-Qs. Table S2: Optimized multiple reaction monitoring parameters of PPDs and PPD-Qs. Table S3: The instrument detection limits (IDLs) and method detection limits (MDLs) for PPDs and PPD-Qs. Table S4: The recoveries of spiked surrogate standard (20 ng, ^13^C_6_-6PPD-Q). Figure S1: The exemplary chromatograms of 6PPD (7.61min). Figure S2: The exemplary chromatograms of IPPD (5.83 min). Figure S3: The exemplary chromatograms of CPPD (7.24 min). Figure S4: The exemplary chromatograms of DPPD (9.93 min). Figure S5: The exemplary chromatograms of DNPD (10.96 min). Figure S6: The exemplary chromatograms of 77PD (9.30 min). Figure S7: The exemplary chromatograms of DTPD (10.72min). Figure S8: The exemplary chromatograms of 6PPDQ (9.95 min). Figure S9: The exemplary chromatograms of IPPDQ (8.44 min). Figure S10: The exemplary chromatograms of CPPDQ (9.69 min). Figure S11: The exemplary chromatograms of DPPDQ (9.09 min). Figure S12: The exemplary chromatograms of 77PDQ (11.30 min). Figure S13. The exemplary chromatograms of DTPDQ (9.70 min).
