# Supplementary material for: Occurrence of Substituted p-Phenylenediamine Antioxidants and Their Quinone Derivatives in the Pearl River Estuary Water System, China
Source: Toxics. 2026 Apr 23;14(5):356. doi: 10.3390/toxics14050356 (PMC13211082; doi:10.3390/toxics14050356)
Supplement: Supplementary file 1 [file toxics-14-00356-s001.zip › toxics-4265099-supplementary.pdf]

Supplementary Materials

Table S1. Full names, CAS numbers, molecular formulas, structures and physicochemical properties of PPDs and PPD-Qs.

Compound	Full name	CAS No.	Molecular formula	Structure	Log K_{ow}	Water solubility (mg/L, 25°C)
6PPD	*N*-(1,3-dimethylbutyl)-*N'*-phenyl-*p*-phenylenediamine	793-24-8	$C_{18}H_{24}N_2$	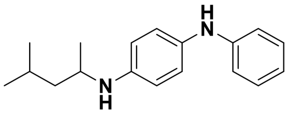	4.47	2.84
IPPD	*N*-isopropyl-*N'*-phenyl-*p*-phenylenediamine	101-72-4	$C_{15}H_{18}N_2$	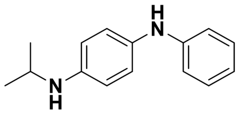	3.28	50.3
CPPD	*N*-phenyl-*N'*-cyclohexyl-*p*-phenylenediamine	101-87-1	$C_{18}H_{22}N_2$	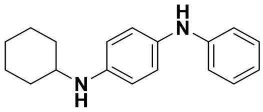	4.64	2.08
77PD	*N*,*N'*-bis(1,4-dimethylpentyl)-*p*-phenylenediamine	3081-14-9	$C_{20}H_{36}N_2$	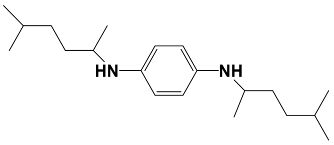	6.30	0.05
DPPD	*N*,*N'*-diphenyl-*p*-phenylenediamine	74-31-7	$C_{18}H_{16}N_2$	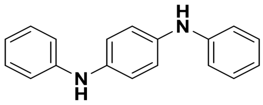	4.93	1.28
DNPD	*N*,*N'*-di-2-naphthyl-*p*-phenylenediamine	93-46-9	$C_{26}H_{20}N_2$	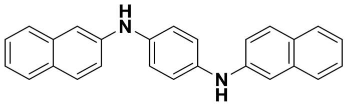	6.39	0.00145
DTPD	*N*,*N'*-bis(methylphenyl)-1,4-benzenediamine	27417-40-9	$C_{20}H_{20}N_2$	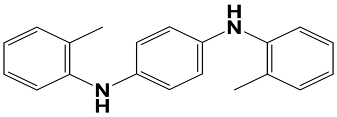	5.13	0.59
6PPD-Q	2-((4-methylpentan-2-yl)amino)-5-(phenylamino)cyclohexa-2,5-diene-1,4-dione	2754428-18-5	$C_{18}H_{22}N_2O_2$	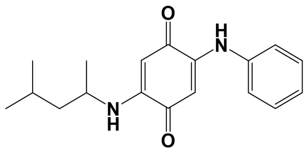	3.98	51.3
IPPD-Q	2-(isopropylamino)-5-(phenylamino) cyclohexa-2,5-diene-1,4-dione	68054-73-9	$C_{15}H_{16}N_2O_2$	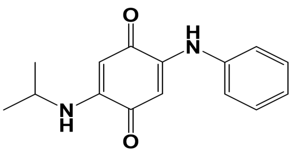	2.58	1400
CPPD-Q	2-(cyclohexylamino)-5-(phenylamino) cyclohexa-2,5-diene-1,4-dione	68054-78-4	$C_{18}H_{20}N_2O_2$	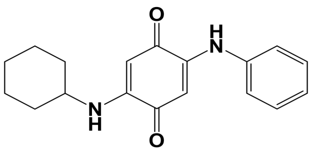	3.94	56.9

77PD-Q	2,5-bis((5-methylhexan-2-yl)amino) cyclohexa-2,5-diene-1,4-dione	NA	$C_{20}H_{34}N_2O_2$	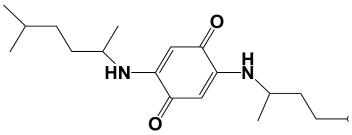	5.47	1.66
DPPD-Q	2,5-bis(phenylamino)cyclohexa-2,5-diene-1,4-dione	3421-8-7	$C_{18}H_{14}N_2O_2$	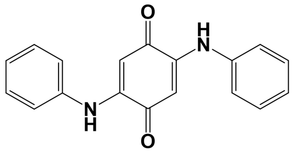	3.46	15.4
DTPD-Q	2,5-bis(o-tolylamino)cyclohexa-2,5-diene-1,4-dione	252950-56-4	$C_{20}H_{18}N_2O_2$	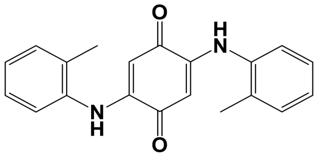	4.56	1.22

Table S2. Optimized multiple reaction monitoring parameters of PPDs and PPD-Qs.

Compound	Precursor ion (m/z)	Production ion (m/z)	Collision (eV)	Cone (V)	Retention time (min)
6PPD	269.2	184.1	30	30	7.61
		212.1	20	30	
IPPD	227.1	184.1	30	30	5.83
		212.1	20	30	
CPPD	267.2	93.1	30	30	7.24
		185.1	15	30	
77PD	305.3	206.1	18	30	9.30
		135.0	30	30	
DPPD	261.1	168.1	35	30	9.93
		184.1	30	30	
DNPD	361.2	234.1	30	30	10.96
		218.1	40	30	
DTPD	289.2	198.1	25	30	10.72
		183.1	30	30	
6PPD-Q	299.2	241.1	30	30	9.95
		215.1	18	30	
IPPD-Q	257.1	187.1	25	30	8.44
		215.1	15	30	
CPPD-Q	297.2	215.1	18	30	9.69
		187.1	25	30	
77PD-Q	335.2	237.1	20	30	11.30
		167.1	30	30	
DPPD-Q	291.1	144.1	30	30	9.09
		263.1	20	30	
DTPD-Q	319.1	184.1	20	30	9.70
		212.1	20	30	

Table S3. The instrument detection limits (IDLs) and method detection limits (MDLs) for PPDs and PPD-Qs.

Compound	IDLs (ng/mL)	MDLs	
		Water (ng/L)	Sediment (ng/g dw)
6PPD	0.015	0.008	0.004
IPPD	0.019	0.009	0.006
CPPD	0.011	0.002	0.001
77PD	0.054	0.011	0.003
DPPD	0.049	0.010	0.002
DNPD	0.032	0.006	0.002
DTPD	0.057	0.011	0.003
6PPD-Q	0.016	0.003	0.001
IPPD-Q	0.019	0.004	0.001
CPPD-Q	0.017	0.003	0.001
77PD-Q	0.018	0.004	0.001
DPPD-Q	0.034	0.007	0.002
DTPD-Q	0.012	0.002	0.001

Table S4. The recoveries of spiked surrogate standard (20 ng, $^{13}C_6$-6PPD-Q).

Site	Spiked surrogate standard (%)	
	in water	in sediment
1	95.9	/
2	75.6	/
3	76.1	83.8
4	97.2	/
5	100.6	85.3
6	98.6	/
7	80.4	/
8	81.5	/
9	65.4	92.0
10	90.6	72.6
11	55.3	/
12	79.8	/
13	84.9	/
14	71.7	/
15	81.6	/
16	67.7	/
17	101.3	/
18	99.4	91.3
19	64.7	/
20	72.4	/
21	75.5	/
22	69.2	/
23	97.3	/

24	78.3	/
25	71.7	/
26	62.9	/
27	87.5	/
28	66.2	/
29	55.2	/
30	58.1	/

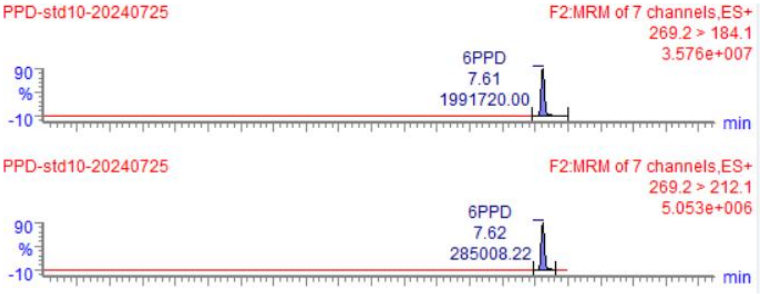

Figure S1. The exemplary chromatograms of 6PPD (7.61min).

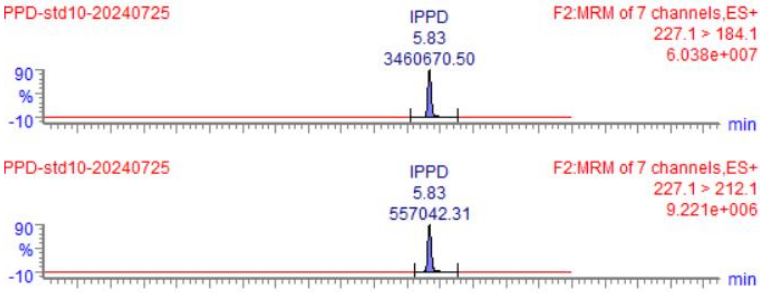

Figure S2. The exemplary chromatograms of IPPD (5.83min).

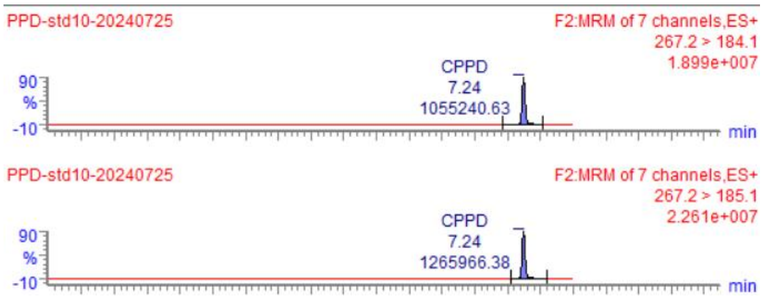

Figure S3. The exemplary chromatograms of CPPD (7.24min).

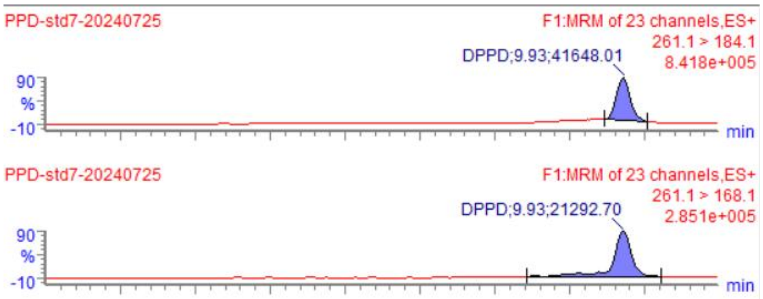

Figure S4. The exemplary chromatograms of DPPD (9.93min).

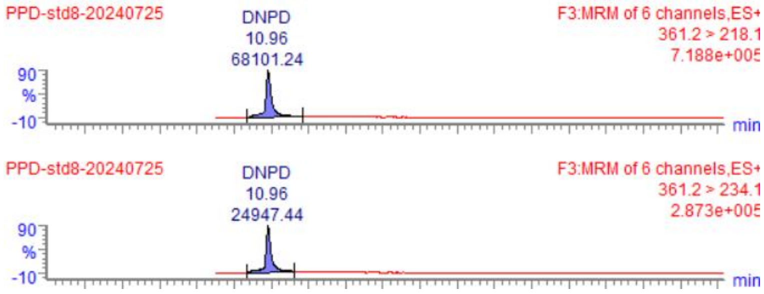

Figure S5. The exemplary chromatograms of DNPD (10.96min).

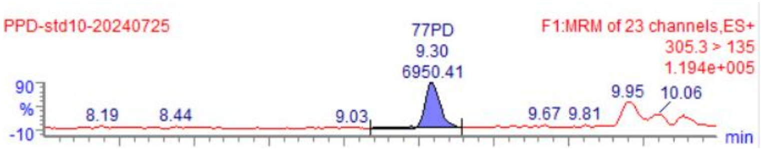

Figure S6. The exemplary chromatograms of 77PD (9.30min).

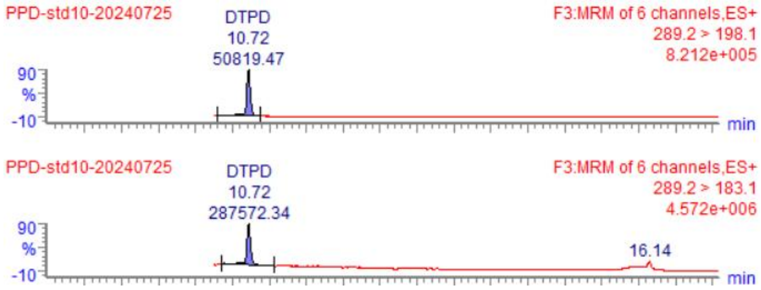

Figure S7. The exemplary chromatograms of DTPD (10.72min).

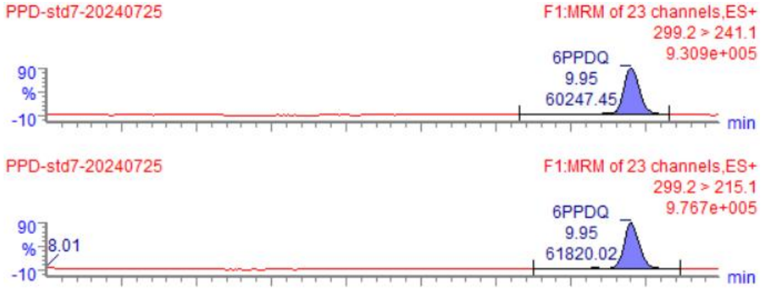

Figure S8. The exemplary chromatograms of 6PPDQ (9.95min).

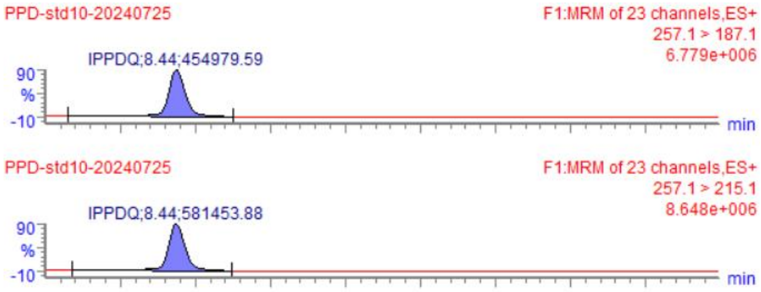

Figure S9. The exemplary chromatograms of IPPDQ (8.44min).

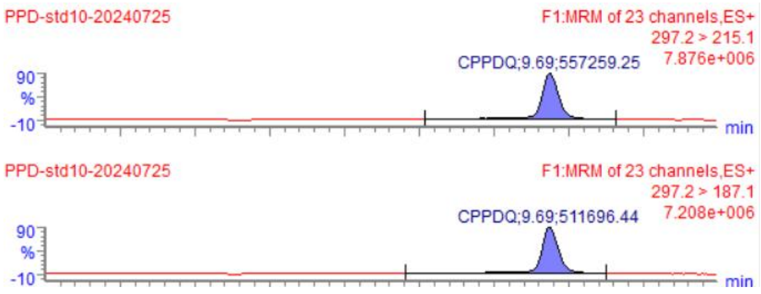

Figure S10. The exemplary chromatograms of CPPDQ (9.69min).

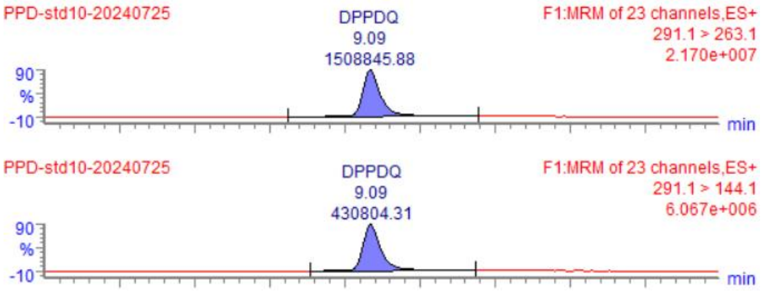

Figure S11. The exemplary chromatograms of DPPDQ (9.09min).

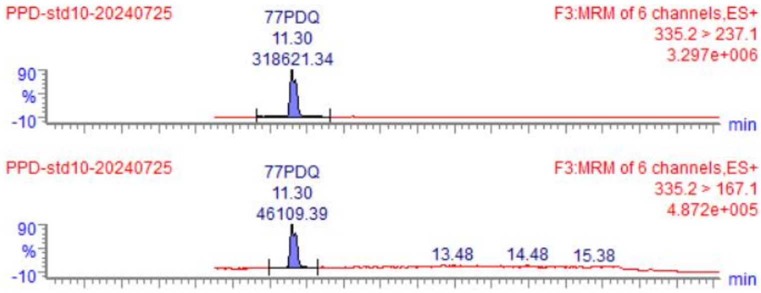

Figure S12. The exemplary chromatograms of 77PDQ (11.30min).

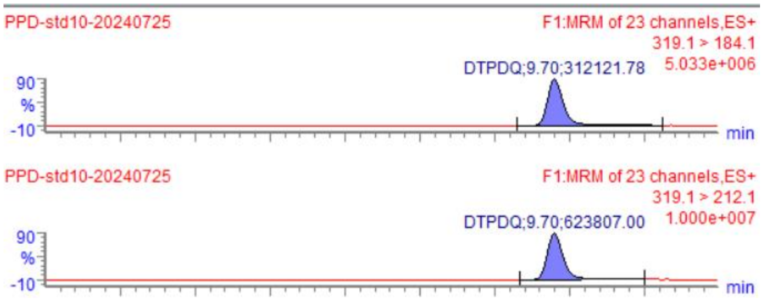

Figure S13. The exemplary chromatograms of DTPDQ (9.70min).
